# Repositioning an Immunomodulatory Drug Vidofludimus as a Farnesoid X Receptor Modulator With Therapeutic Effects on NAFLD

**DOI:** 10.3389/fphar.2020.00590

**Published:** 2020-05-14

**Authors:** Yanlin Zhu, Shuangshuang Xu, Yi Lu, Yijuan Wei, Benqiang Yao, Fusheng Guo, Xing Zheng, Yumeng Wang, Ying He, Lihua Jin, Yong Li

**Affiliations:** ^1^State Key Laboratory of Cellular Stress Biology, School of Life Sciences, Innovation Center for Cell Signaling Network, Xiamen University, Xiamen, China; ^2^Xiamen Key Laboratory of Neonatal Diseases, Xiamen Children’s Hospital, Xiamen, China; ^3^Laboratory Animal Center, Xiamen University, Xiamen, China; ^4^Department of Diabetes Complications and Metabolism, Diabetes and Metabolism Research Institute, Beckman Research Institute, City of Hope National Medical Center, Duarte, USA

**Keywords:** inflammatory bowel disease (IBD), nuclear receptor, ligand-binding protein, liver metabolism, drug discovery, drug screening, crystal structure, receptor

## Abstract

Non-alcoholic fatty liver disease (NAFLD) has become the most common chronic liver disorder, and yet with no pharmacological treatment approved worldwide. The repositioning of old drugs provides a safe approach for drug development. Vidofludimus, an inhibitor for dihydroorotate dehydrogenase (DHODH) for the treatment of autoimmune disorders, is herein uncovered as a novel modulator for farnesoid X receptor (FXR) by biochemical and crystallographic analysis. We further revealed that vidofludimus exerts *in vivo* therapeutic effects on dextran sodium sulfate (DSS)-induced colitis in an FXR-dependent manner. Notably, vidofludimus also possesses remarkable beneficial effects in reducing NAFLD by targeting FXR, which may represent a unique approach in developing the treatment for NAFLD. Our findings not only reveal a promising template for the design of novel FXR ligands in treating autoimmune disorders, but also uncover a novel therapeutic effect for vidofludimus on NAFLD based on the newly established relationships among drugs, targets, and diseases.

## Highlights

The ligands that regulate FXR activity are promising therapeutic agents for several diseases. However, the clinical applications of FXR ligands remain much less developed. This study identifies vidofludimus as a ligand for nuclear receptor FXR and showed therapeutic effects on colitis depending on FXR. Furthermore, vidofludimus has been repurposed to treat fatty liver by targeting FXR based on the new established relationships among drugs, targets, and diseases.

## Introduction

Non-alcoholic fatty liver disease (NAFLD), caused by accumulation of abnormal amounts of fat in the liver due to causes other than alcohol, has become the most common liver disorder in developed countries (Pappachan et al., 2017). Among a variety of hepatic dysregulation caused by NAFLD, non-alcoholic steatohepatitis (NASH) is the extreme form with features of inflammation and fibrosis (Ratziu, 2015; Chao et al., 2016). Despite extensive studies and investment, no pharmacological treatment has been approved worldwide so far for the effective control of NAFLD. Nuclear farnesoid X receptor (FXR) plays pivotal roles in regulating inflammatory processes and metabolism (Flatt et al., 2009; Pellicciari et al., 2016; Tully et al., 2017; Sepe et al., 2018) and, thus, has become a hot target for drug development. With the development of obeticholic acid (OCA) (Pellicciari et al., 2002), a semi-synthetic bile acid analogue and a ligand for FXR, in clinical trials for NASH, FXR has emerged as an attractive drug target for NAFLD.

Several lines of evidences have suggested the importance of FXR in the management of NAFLD (Yang et al., 2010; Lefebvre and Staels, 2014; Lu et al., 2014; Xiong et al., 2014; Carr and Reid, 2015; Jin et al., 2015; Tully et al., 2017; Xiao et al., 2017). The reduced expression of FXR has been observed in livers of obese rodents and NAFLD patients (Yang et al., 2010; Lu et al., 2014; Xiong et al., 2014). In addition, FXR knockout led to hepatic steatosis in mice, which were associated with the induction of inflammatory genes, such as IFNγ and TNFα (Sinal et al., 2000; Zhang et al., 2006; Wang et al., 2008; Mencarelli et al., 2009). On the other hand, activation of FXR suppressed the induction of inflammation genes in the liver and intestine, possibly by repression of NF-kB signaling (Wang et al., 2008; Vavassori et al., 2009; Gadaleta et al., 2011a; Gadaleta et al., 2011b). Interestingly, treatment with FXR agonist effectively alleviates fat accumulation in liver, suggesting the therapeutic effects of FXR agonists in NAFLD (Zhang et al., 2006; Haczeyni et al., 2017; Tully et al., 2017; Xiao et al., 2017). Indeed, a series of FXR ligands have been executed in clinical trials, such as OCA (Intercept), LJN452 (Novartis), GS9674 (Gilead), and EDP305 (Enanta), as treatments for FXR-mediated diseases, including primary biliary cholangitis (PBC), NASH, primary sclerosing cholangitis (PSC), or biliary atresia. The development of these candidates strongly proves the feasibility of FXR as a target for metabolic diseases. However, despite much encouraging progress in applying FXR ligands in clinical trials (Mudaliar et al., 2013; Neuschwander-Tetri et al., 2015; Younossi et al., 2019), various adverse side effects associated with FXR ligands limited their further development and clinical application. So far, OCA is the only FDA-designated breakthrough therapy in development for NASH with compensated cirrhosis. As such, a new drug-design strategy for the identification of novel FXR ligands is still an utmost need to yield more efficacious FXR-targeted drugs with reduced adverse effects.

Repositioning of drugs through identification of their potentials for functionally interacting with unintended targets, offers an increasing productivity than traditional drug development (Hu et al., 2014; Wang et al., 2014; Bao et al., 2016; Krishnan et al., 2018). This strategy can not only improve the optimization of current drugs, but also lead to new therapeutic switching. In this study, we report the identification of an immunomodulatory drug vidofludimus [2-(3-Fluoro-3′-methoxybiphenyl-4-ylcarbamoyl)-cyclopent-1-enecarboxylic acid (4SC-101)] as a novel modulator for FXR with a therapeutic potential in the treatment of NAFLD.

## Materials and Methods

### Protein Preparation

The human FXR ligand binding domain (LBD, residues 243–472) was expressed as N-terminal 6×His fusion protein from the expression vector pET24a (Novagen, Madison, WI, USA). BL21 (DE3) cells transformed with expression plasmids were grown in LB broth at 25°C to an OD_600_ of ~1.0 and induced with 0.1 mM isopropyl 1-thio-β-d-galactopyranoside (IPTG) at 16°C. Cells were harvested and sonicated in 200 mL extraction buffer (25 mM Tris, pH 7.5, 300 mM NaCl, 10% glycerol, and 25 mM imidazole) per 6 L of cells. The lysate was centrifuged at 20,000 rpm for 30 min at 4°C, and the supernatant was loaded on a 5-mL NiSO_4_-loaded HiTrap HP column (GE Healthcare, Piscataway, NJ, USA). The column was washed with extraction buffer, and the protein was eluted with a gradient of 25 to 500 mM imidazole. The FXR LBD was further purified by gel filtration using a HiLoad 26/600 Superdex 200 column (GE Healthcare). The FXR LBD was complexed with a five-fold molar excess of vidofludimus (TargetMol, China) and a two-fold molar of a SRC2-3 peptide (ENALLRYLLDKD) before filter concentration.

### Coregulator Binding Assays

The binding of the various coregulator peptide motifs to FXR LBD in response to ligands was determined by AlphaScreen™ (Amplified Luminescent Proximity Homogeneous Assay Screen) assays using a hexahistidine detection kit from PerkinElmer as described before (Jin et al., 2013). The experiments were conducted with approximately 20 to 40 nM receptor LBD and 20-100 nM biotinylated cofactor peptides in the presence of 5 µg mL^−1^ donor and acceptor beads in an Alpha buffer containing 50 mM MOPS, 50 mM NaF, 0.05 mM CHAPS, and 0.1 mg mL^−1^ bovine serum albumin, all adjusted to a pH of 7.4. The peptides with an N-terminal biotinylation are listed below.

SRC1-2, SPSSHSSLTERHKILHRLLQEGSP;
SRC2-3, QEPVSPKKKENALLRYLLDKDDTKD;
SRC3-3, PDAASKHKQLSELLRGGSG;
NCOR-1, GQVPRTHRLITLADHICQIITQDFARNQ;
NCOR-2, GHSFADPASNLGLEDIIRKALMGSF.

### Drug Screening by AlphaScreen Assay

We first constructed a compound library which contains 576 compounds in clinical phase purchased from TargetMol (Shanghai, China), 576 compound monomers extracted from Chinese herbal medicine, 960 compound monomers extracted from marine products, and 1152 synthetic compounds, totally 3264 compounds. The primary screen was carried out using 50 nM H6-FXR LBD, 100 nM Biotin-SRC1-2, 10 μM compounds, and 5 μg mL^−1^ donor and acceptor beads in the Alpha buffer, using the same method as in coregulator binding assays. We first got 13 candidate compounds. We next retested the 13 compounds by four repeated experiments and excluded 8 false-positive compounds. Then, we retested the five remaining compounds with seven concentrations dose–response and got the most probably FXR agonist candidate.

### Reporter Assay

COS-7 or 293T cells were maintained in Dulbecco’s Modified Eagle Medium (DMEM) containing 10% fetal bovine serum (FBS) at 37°C in 5% CO_2_ and were transiently transfected using Lipofectamine 2000 (Invitrogen). Before transfection, 24-well plates were plated with of 5 x 10^4^ cells per well. For full-length receptor assay system, cells were co-transfected with 0.2 µg pCMV-hFXR, 0.2 µg EcRE-Luc, and 0.05 µg renilla, with or without 1.2 µg pCMV-hRXR. For pBind reporter assay system, cells were co-transfected with 0.2 µg pBind-hFXR LBD and 0.2 µg pG5-luc. Additionally, we set another control which was co-transfected with 0.2 µg pcDNA-hPPARγ, 0.2 µg PPRE-Luc, and 0.05 µg renilla. 1 µM to 5 µM ligands were added 6 h after transfection. DMSO was used as a negative control, 1 µM OCA was used as the positive control for FXR, and 1 µM rosiglitazone was used as the positive control for PPARγ. Cells were harvested 24 h later for luciferase assays with a dual-luciferase reporter assay system (Promega). The luciferase activities were normalized to Renilla cotransfected as an internal control.

### Crystallization and Structure Determination

The crystals of FXR/vidofludimus complex were grown at room temperature in hanging drops containing 1.0 μl of the ligand-protein solutions and 1.0 μl of well buffer containing 0.2 M lithium chloride and 25% w/v polyethylene glycol 6000. The crystals were directly flash frozen in liquid nitrogen for data collection. The observed reflections were reduced, merged, and scaled with DENZO and SCALEPACK in the HKL2000 package (Otwinowski and Minor, 1997). The structures were determined by molecular replacement in the CCP4 suite. Manual model building was carried out with COOT (Emsley and Cowtan, 2004), followed by Refmac5 refinement in the CCP4 suite.

### Induction of Colitis and Animal Treatment

10-week-old male wild-type C57BL/6J (WT) and homozygous FXR deficient (FXR KO) mice in C57BL/6 background (Sinal et al., 2000) were maintained under environmentally controlled conditions with free access to standard chow diet and water. Animal experiments were conducted in the barrier facility of the Laboratory Animal Center, Xiamen University, approved by the Institutional Animal Use and Care Committee of Xiamen University, China. Littermates of the same sex were randomly assigned to experimental groups (n = 6 per group). For induction of colitis, mice were fed with 2.5% (w/v) dextran sodium sulfate (DSS) in drinking water for 10 days. Vidofludimus (20 mg/kg/day) was administrated orally from 3 days before DSS treatment, and continued until the end of the experiments. Mice were killed by cervical dislocation. 1.5 cm of distal colonic sections were fixed in 4% paraformaldehyde, embedded in paraffin, sectioned, and stained with hematoxylin and eosin (H&E). Histopathological scoring was performed by using an established semi-quantitative score ranging from 0 to 6 based on infiltration of inflammatory cells and epithelial damage. Depletion of goblet cells was scored using a scoring index from 0 to 4 as described previously (Gadaleta et al., 2011a).

### NAFLD Model Experiments

10-11 weeks old male obese Lep^ob/ob^ C57BL/6 (ob/ob) mice were purchased from the Model Animal Research Center of Nanjing University, China. Mice were randomly assigned to experimental groups. Mice were intraperitoneally (i.p.) injected with either vehicle (40% of 2-hydroxypropyl-β-cyclodextrin (HBC), Sigma, USA) or vehicle containing vidofludimus (10 mg/kg) once daily for 14 days(n = 6 per group). After fasting for 6 h, mice were weighed and sacrificed by cervical dislocation. Liver histology characterization was analyzed by H&E staining with paraffin-embedded sections. Fresh liver tissues were embedded in optimum cutting temperature compound (OCT) and cryo-sectioned. The sections were fixed in 4% paraformaldehyde in PBS, and stained with 0.3% oil red O. Other tissues were collected and frozen in liquid nitrogen for use, and plasma were collected to measure metabolite parameters.

### Cells

Human hepatocellular carcinoma cell line HepG2 was maintained in DMEM containing 10% FBS. Primary mouse embryonic fibroblast (MEF) cells were extracted from fetus of 14 d–16 d WT and FXR KO pregnant female mice and cultured in DMEM with 10% FBS.

### Western Blotting

HepG2 cells or MEFs were treated with vidofludimus for 1 h before exposed to TNFα (20 ng/mL) for additional 30 min (for HepG2 cells) or 1 h (for MEFs). Lysates prepared were analyzed with anti-IκBα, anti-phospho IKKα/β (Cell Signal Technology), and anti-β-actin (Cell Signal Technology) antibodies by Western blotting. Nuclear extracts from the distal colon or treated MEFs were used for assessing p65 protein level using p65 antibody (Santa Cruz) with anti-Lamin B1 (Proteintech) as internal controls. Western blotting was performed as standard procedures.

### Quantitative Real-Time PCR (qPCR)

HepG2 cells were co-transfected with plasmids pCMX-FXR (200 ng) or pCMX-Empty Vector (200 ng) and treated with vehicle (DMSO), 1 μM OCA, 5 ng/mL TNFα, TNFα plus OCA, or 5 μM vidofludimus in the presence or absence of TNFα for 24 h. RNA was isolated using a Tissue RNA kit (Omega Bio-Tek, GA), and was reverse transcribed using a TAKARA reverse transcription kit. qPCR were performed on a CFX96™ Real-Time PCR Detection System (Bio-Rad) using SYBR Premix Ex Taq™ (TAKARA). Relative mRNA expression levels were normalized to B2M or 36B4 gene levels. The sequences of primers used were listed in **Table S2**.

### Statistical Analysis

All values are mean ± s.e.m. Data in Figure 6 were analyzed by Student’s t test (two-tailed). The statistical significance of the differences of data in Figure 4 and **Figure S5** were determined using one-way ANOVA with Dunnett’s multiple comparisons test. A P value of <0.05 was considered significant.

### Data and Software Availability

The crystal structure of FXR in complex with vidofludimus has been deposited in the PDB under ID code 5y1j. For statistics, see **Table S1**.

## Results

### Identification of an Immunomodulatory Drug Vidofludimus as an FXR Modulator

As a ligand-regulated nuclear receptor, the activity of FXR is mediated through ligand-dependent recruitment or release of specific co-regulators, including coactivators, such as the steroid receptor coactivators (SRCs), and corepressors, such as NCoR (Lefebvre et al., 2009). In search for novel modulators for FXR, we screened a compound library using an AlphaScreen assay, which determines the efficacy of small molecules in influencing binding affinity of nuclear receptors with co-regulator peptides (Jin et al., 2013).

**Figure 1:**
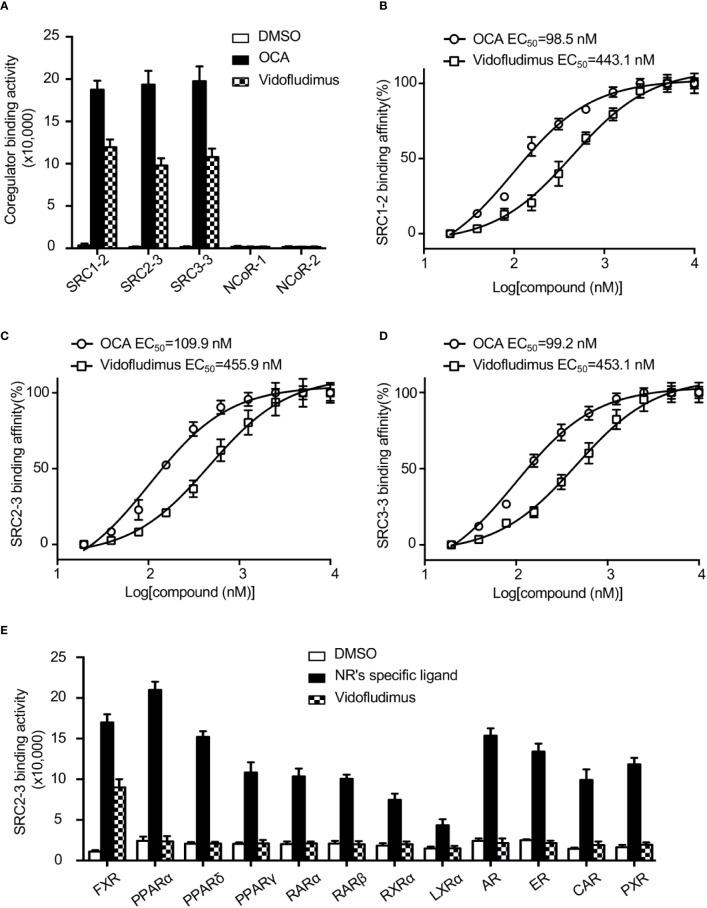
Vidofludimus, an anti-inflammatory drug in clinical phase, was uncovered as a novel FXR ligand. **(A)** Various co-regulator binding motifs bind to FXR in response to 1 µM vidofludimus or OCA by AlphaScreen assay. **(B–D)** Dose–response curves and EC_50_s for vidofludimus in inducing FXR to recruit coactivator motifs by AlphaScreen assay. The peptide sequences are listed in Experimental Section. **(E)** Purified nuclear receptor LBD proteins, including His × 6 tagged FXR, PPARα, PPARδ, PPARγ, RARα, RARβ, RXRα, and LXRα, and GST-tagged AR, ER, CAR and PXR were used in the assay to test the abilities of recruiting biotin-labeled SRC2-3 peptide induced by DMSO, 1 μM vidofludimus or ligands specific for each receptor: FXR, 1 μM OCA; PPARα, 1 μM GW590735; PPARδ, 1 μM GW0472; PPARγ, 1 μM rosiglitazone; RARα and RARβ, 1 μM all-trans-retinoic acid; RXRα, 1 μM 9-cis-retinoic acid; LXRα, 1 μM 22(R)-hydroxycholesterol; AR, 0.1 μM dihydrotestosterone; ER, 0.1 μM estradiol; PXR, 1 μM rifampicin; CAR, 1 μM CITCO. Values are the means ± s.e.m. of three independent experiments.

The results revealed that vidofludimus (SC12267 or 4sc-101) (**Figure S1**), a drug in clinical phase with a chemical structure distinct from reported FXR ligands such as GW4064, CDCA, and OCA, strongly promoted the interaction of FXR with various coactivator LXXLL motifs from the family of SRCs (SRC1, SRC2, and SRC3), with a less extent than that of OCA, while without impacting the recruitment of co-repressor motifs from NcoR (**Figure 1A**). Furthermore, full-dose curves revealed that vidofludimus activated FXR in a concentration dependent manner with an EC_50_ of about 450 nM in inducing the recruitment of various coactivator LXXLL motifs (**Figures 1B–D**), suggesting that vidofludimus is a highly potent FXR ligand. We further performed an AlphaScreen assay to test the selectivity of vidofludimus against other nuclear receptors. The results revealed that vidofludimus selectively activated the coactivator recruitment by FXR, with no impacts on other nuclear receptors tested, including peroxisome proliferator-activated receptor (PPAR) α, δ, and γ, retinoic acid receptor (RAR) α and β, retinoid X receptor (RXR) α, liver X receptor (LXR) α, androgen receptor (AR), estrogen receptor (ER), progesterone receptor (PXR), and constitutive androstane receptor (CAR) (**Figure 1E**). These results suggest that vidofludimus is a selective ligand of nuclear receptor FXR.

### Structural Determination of the FXR LBD in Complex With Vidofludimus

**Figure 2:**
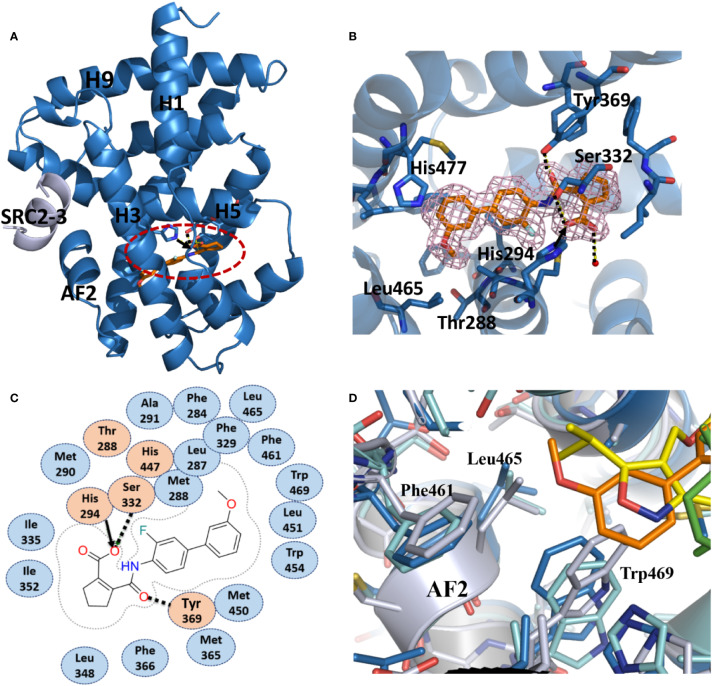
Recognition of vidofludimus by FXR. **(A)** The structure of vidofludimus bound with FXR LBD in carton representation (PDB ID 5y1j). FXR LBD is colored in dark blue and the SRC2-3 motif is in gray. The bound vidofludimus drug is shown in stick representation with carbon, nitrogen, and oxygen atoms depicted in orange, blue, and red, respectively. **(B)** 2Fo-Fc electron density map (1.0σ) showing the bound vidofludimus and the surrounding FXR residues. **(C)** Schematic representation of FXR-vidofludimus interaction. The residues labeled orange are polar, while the residue in blue indicates hydrophobic one. Hydrogen bonds are indicated by arrows from proton donors to acceptors, and the charge interaction was indicated by solid lines with arrow. **(D)** The hydrophobic environment that contacts ligands for stabilizing the active conformation of helix 12 of FXR, where ligand vidofludimus is in orange, GW4064 (PDB ID 3dct) is in yellow, and OCA (PDB ID 1ot7) is in green.

**Figure 3:**
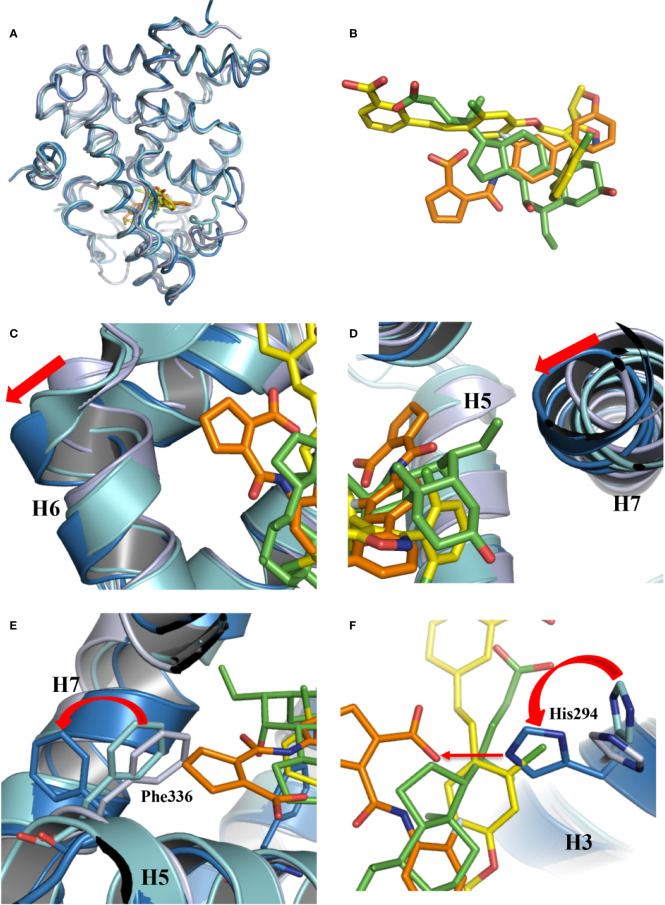
Conformational changes of FXR induced by the binding of vidofludimus. **(A)** Overlays of the structures of vidofludimus (orange)-bound FXR (dark blue) (PDB ID 5y1j) with GW4064 (yellow)-bound FXR (cyan) (PDB ID 3dct), and OCA (green)-bound FXR (gray) (PDB ID 1ot7). **(B)** Superposition of vidofludimus (orange) with GW4064 (yellow) and OCA (green). **(C–F)** Overlays of the FXR-vidofludimus structure (dark blue) with the FXR-GW4064 (cyan) and FXR-OCA (gray), where ligands vidofludimus, GW4064, and OCA are in orange, yellow, and green, respectively.

To unravel the molecular basis for the recognition of FXR by vidofludimus, we performed structural studies on the FXR LBD complexed with vidofludimus. The data and model refinement statistics of the crystal structure are summarized in **Table S1** (PDB ID 5y1j). The structure revealed that the vidofludimus-bound FXR LBD adopts a canonical active conformation in a three-layer helical sandwich arrangement that resembles most agonist-bound nuclear receptor structures (**Figure 2A**). The existence of vidofludimus was apparent from the highly revealing electron density map showed in **Figure 2B**, whose interaction with FXR was stabilized by a combination of hydrogen bonds, charge interactions, and hydrophobic interactions (**Figure 2C**). Superposition of different ligand-bound FXR structures revealed that vidofludimus aligned well with GW4064 (PDB ID 3dct) (Akwabi-Ameyaw et al., 2009) and OCA (PDB ID 1ot7) (Mi et al., 2003) and occupied a similar binding site in the FXR pocket (**Figure 3A**). Notably, the methoxyl group of vidofludimus initiates steric clashes with the hydrophobic environment for stabilizing helix 12 of FXR, partly composed of F461, L465, and W469 on AF2 (**Figure 2D**, **Figure S2**). The conformation of the FXR helix 12, an activation function-2 (AF-2) motif, is critical to the ligand-induced co-regulator recruitment (Jin and Li, 2010). The binding of vidofludimus thus interferes with the configuration of the active conformation of FXR AF2 (**Figure S2D**), resulting in the weaker ability in recruiting coactivator motifs than that of OCA (**Figures 1A–D**).

With a unique molecular structure different from GW4064 and OCA (**Figure 3B**, **Figure S1**), vidofludimus also displays unique interactions with FXR, which induces series of conformational changes of FXR pocket to adapt to the ligand binding. Compared to the FXR bound with GW4064 and OCA, the helix 6 of vidofludimus-bound FXR was shifted outward, while the helix 7 was shifted inward upon the binding of vidofludimus (**Figures 3C, D**). In addition, unlike long acid substituent group in the GW4064, OCA, and CDCA (**Figure S2B**) which insert the polar pocket formed by H1, H5, and H3, the cyclopentene acid group in vidofludimus occupies the hydrophobic pocket formed by H5, H6, and H7(**Figures S2A, C**). Although there are several mechanistic studies that have demonstrated that canonical Arg331 interaction plays an important role in full agonists to FXR, due to classic target genes in hepatocytes capture activation efficacy under treatment of OCA and vidofludimus (**Figure S3**), this polar interaction seem to be not necessary in FXR activation epically in partial agonists and FXR modulators. Moreover, in this binding mode the hydrophobic side chains of F336 shifted outward to make extra space for the binding of vidofludimus (**Figure 3E**), while the polar H294 shifted to initiate a hydrogen bond with vidofludimus (**Figure 3F**, **Figure S2D**). Due to the recent study (Merk et al., 2019), the partial agonism in contrast is conferred by a kink in helix α11 that destabilizes the H11–H12 loop which is a critical determinant for helix α12 orientation. The conformational changes of FXR pocket in H11-H12 loop induced by vidofludimus are more similar to those induced by GW4064 which stabilizes formation of an extended helix α11 and binds the H11-H12 loop; but different with those induced by CDCA and OCA (**Figure S4**). Therefore, vidofludimus cannot induce FXR to recruit NCoR (**Figure 1A**). All these indicate that FXR has a great flexibility to adapt the binding of diverse ligands.

### Vidofludimus Ameliorates Histological Pathology Associated With DSS-Induced Colitis in an FXR-Dependent Manner

**Figure 4:**
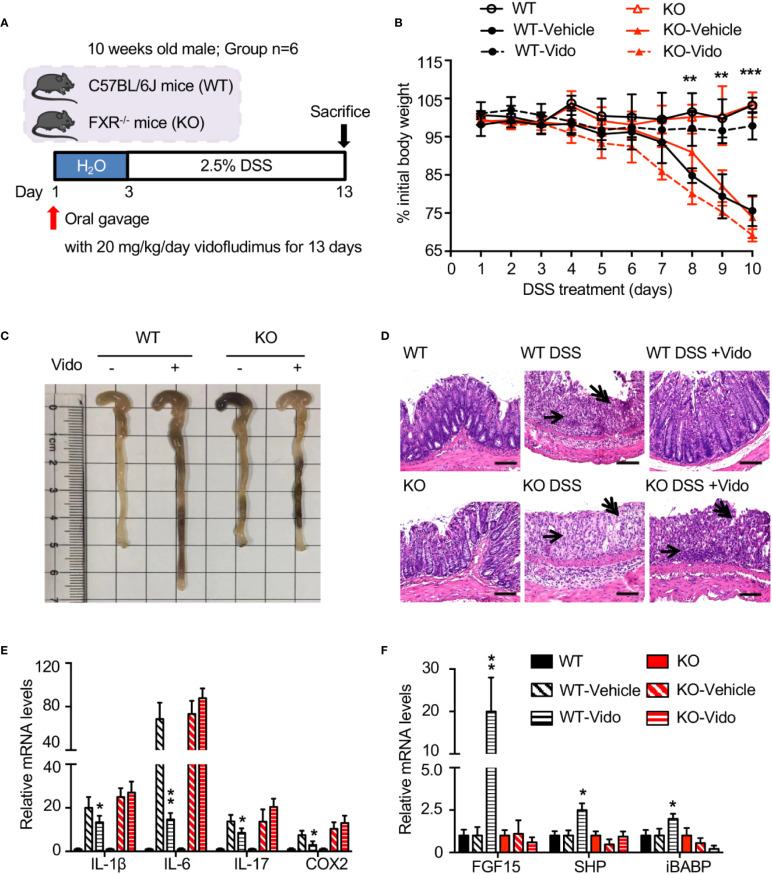
Vidofludimus ameliorates histological pathology associated with DSS-induced colitis in an FXR-dependent manner. **(A)** Mice were treated daily with vehicle or vidofludimus by p.o. gavage for 13 days, with tissues and serum collected 6 h after the final treatment. Mice (WT, KO) treated with PBS instead of DSS were used as controls. **(B)** Percentage of initial body weight during DSS treatment. **(C)** Representative images for colon of wild-type (WT) and FXR knockout (KO) mice; **(D)** Representative images for H&E-stained colon sections. DSS-treated WT mice that received vidofludimus (Vido) were protected from DSS-induced colitis. Single-headed arrows point to inflammatory infiltrates and double-headed arrows to epithelial degeneration. Scale bars, 100 µm. **(E)** qRT-PCR analysis of inflammatory genes in colon homogenates from WT and KO mice administered with DSS. **(F)** qRT-PCR analysis of FXR target genes in ileum homogenates from WT and KO mice administered with DSS. Values are mean ± s.e.m of 6 mice. *p < 0.05, **p < 0.01, ***p < 0.001 versus vehicle-treated mice.

Vidofludimus, an inhibitor for dihydroorotate dehydrogenase (DHODH), is in clinical trials for the treatment of autoimmune disorders such as inflammatory bowel disease (IBD) (Fitzpatrick et al., 2010; Herrlinger et al., 2013). The results from previous work suggest that there may be alternative pathways in addition to DHODH for the anti-inflammation effects regulated by vidofludimus (Fitzpatrick et al., 2012). We wondered whether the functional roles of FXR are involved in the IBD therapy through vidofludimus. As expected, vidofludimus significantly ameliorated DSS-induced colitis as assessed by significantly reduced body weight loss, prevented colonic shortening, decreased histological scores, and disease activity index (DAI) scores in WT mice (**Figures 4A–C**, **Figure S5**). Interestingly, the IBD therapeutic effects of vidofludimus were not observed in FXR KO mice, suggesting the functional importance of FXR in the pharmacological pathway of this immunomodulatory drug candidate. Distal colonic sections from vidofludimus-treated FXR KO mice further revealed multifocal inflammatory cell infiltration and edema with crypt and epithelial cell destruction and ulceration (**Figure 4D**). By contrast, no mucosal inflammation was observed in colonic sections of vidofludimus-treated WT mice (**Figure 4D**). In DSS-treated WT mice, but not FXR KO mice, vidofludimus significantly decreased colonic mRNA expression of the pro-inflammatory genes interleukin (IL)-1β, IL-6, IL-17, and prostaglandin-endoperoxide synthase 2 (COX-2) (**Figure 4E**). Consistent with these observations, chronic per os (p.o.) treatment with vidofludimus induced multiple intestinal FXR target genes, including fibroblast growth factor 15 (FGF15), small heterodimer partner (SHP), and ileal bile acid binding protein (IBABP) in WT mice, but failed to activate their expression in FXR KO mice (**Figure 4F**). These data indicate that vidofludimus exerts the therapeutic effects on DSS-induced colitis in an FXR-dependent manner.

### Vidofludimus Mediates the Anti-Inflammatory Effects by Targeting FXR

**Figure 5:**
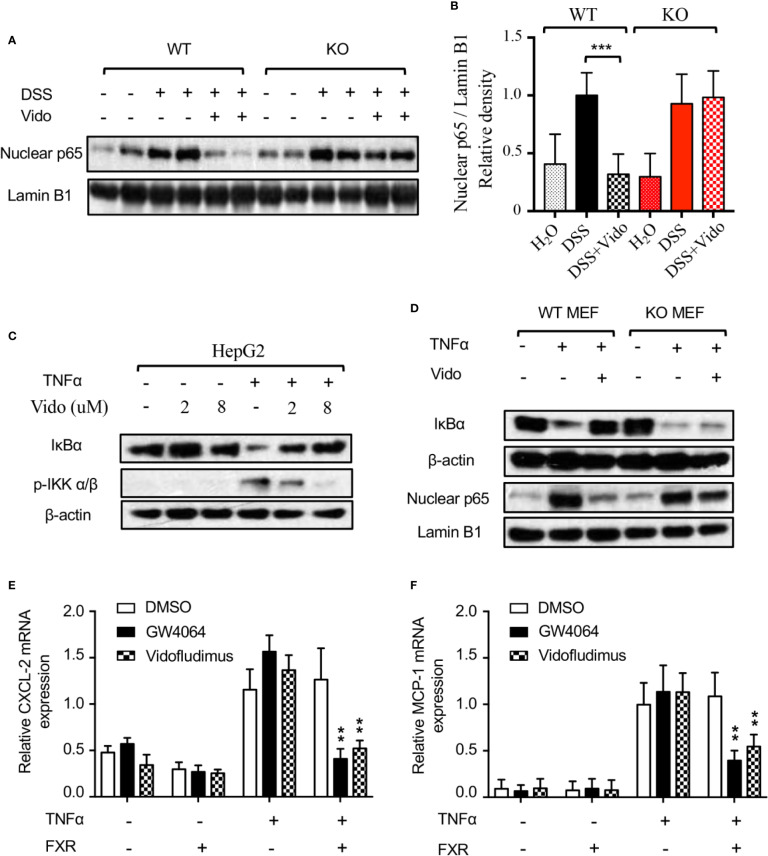
Vidofludimus treatment blocked nuclear translocation of p65 by suppressing IKK-IκB-NF-κB pathway. **(A, B)** Western blotting analysis of nuclear p65 from colon of WT and FXR KO mice treated with DSS and/or vidofludimus. The relative density of the western blotting bands of **(A)** is shown in **(B)**. For **(B)**, values are mean ± s.e.m. ***p < 0.005 versus the samples from mice with DSS treatment. **(C)** Inhibition of TNFα-induced IKKα/β phosphorylation and IκBα degradation by vidofludimus. HepG2 cells treated with vidofludimus for 1 h and/or TNFα (20 ng/ml) for additional 30 min were analyzed by western blotting. **(D)** Western blotting analysis of IκBα and nuclear p65 levels in MEFs from WT and FXR KO mice. MEF cells were treated with vidofludimus (5 μM) for 1 h and/or TNFα (20 ng/mL) for additional 1 h. **(E, F)** Cells were transfected with either empty vector or FXR and treated in triplicate with DMSO, GW4064 (1 μM), vidofludimus (5 μM), TNFα (5 ng/mL), GW4064 plus TNFα, or TNFα plus vidofludimus for 24 h. CXCL-2 and MCP-1 mRNA expression was analyzed by qPCR in duplicate. Values are the means ± s.e.m. of three independent experiments. **p < 0.01, versus cells transfected with FXR treated with DMSO plus TNFα.

The nuclear factor (NF)-κB is an important transcriptional factor that regulates the expression of a variety of genes involved in the control of the immune system and inflammatory response. It has been reported that vidofludimus repressed the nuclear protein level of NF-κB p65 subunit stimulated by trinitrobenzene sulfonic acid (TNBS) in rats (Fitzpatrick et al., 2012), for which the mechanism remains unclear. Interestingly, our result revealed that vidofludimus reduced the nuclear protein level of p65 stimulated by DSS by targeting FXR (**Figures 5A, B**), highlighting the important roles of FXR in the actions of vidofludimus.

The nuclear translocation of p65 is determined by the dissociation of the cytometric NF-κB/IκB complex due to the degradation of IκB (Yamamoto and Gaynor, 2004). Stimuli including the TNF-α activate the IKK complex by inducing the phosphorylation of IKK α/β, resulting in the degradation of IκB proteins (Yamamoto and Gaynor, 2004). We then investigated the protein stabilization of IκBα and the upstream phosphorylation of IKK α/β by the vidofludimus treatment in TNFα stimulated HepG2 cells. As shown in **Figure 5C**, TNFα obviously induced the phosphorylation of IKK α/β and lowered the level of IκBα. Furthermore, vidofludimus treatment suppressed the TNFα-induced phosphorylation of IKK α/β, leading to the inhibition of the degradation of IκBα protein in a concentration-dependent manner, which could further capture p65 in the cytoplasm and block the nuclear translocation of p65. Meanwhile, we analyzed NF-κB target gene expression in HepG2 cells. Cells were stimulated with TNFα to induce NF-κB activity. Indeed, NF-κB target genes MCP-1 and CXCL-2 increased upon TNFα stimulation. Co-treatment with vidofludimus or OCA abolished this effect in HepG2 cells transfected with FXR, but not in cells transfected with empty vector control (**Figures 5E, F**), indicating that FXR activation by OCA or vidofludimus blocks NF-κB activity.

To further investigate the role of FXR in this pathway, we isolated MEF cells from WT and FXR KO mice and assessed the IκBα protein level and the nuclear p65 level. As expected, TNFα induced the degradation of IκBα and increased the nuclear p65 level, vidofludimus treatment obviously stabilized IκBα and inhibited p65 protein level in the nucleus of WT MEFs, and both effects were substantially reduced in FXR KO MEFs (**Figure 5D**), which is consistent with the effects of p65 reduction by vidofludimus in vivo (**Figures 5A, B**). Together, our data demonstrate that vidofludimus might ameliorate colitis through the IKK-IκB-NF-κB signaling pathway mediated by FXR, thereby revealing an alternative functional target of this immunomodulatory drug candidate.

### Vidofludimus Reduces Hepatic Steatosis and Inflammation by Targeting FXR in ob/ob Mice

**Figure 6:**
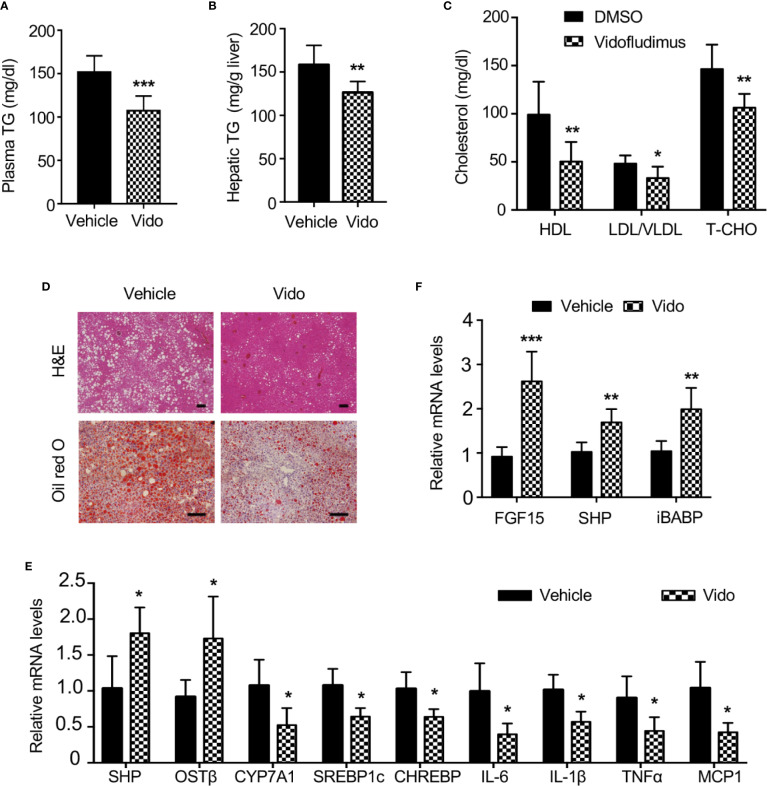
Vidofludimus reduces hepatic steatosis and inflammation in ob/ob mice. 10-11 weeks of male ob/ob mice were intraperitoneally (i.p.) injected with vehicle or vidofludimus (Vido, 10 mg/kg) once daily for 14 days. **(A–C)** Plasma parameters after 6-h fast. TG, triglycerides; TCHO, total cholesterol. **(B)** Hepatic triglyceride (TG) levels. **(D)** Representative images for histological visualization of steatosis in liver sections stained with H&E and oil red O. Scale bars, 100 µm. **(E)** Relative mRNA expression of hepatic FXR target genes and genes involved in lipogenesis and inflammation. **(F)** qPCR analysis of FXR target genes in ileum homogenates. Values are mean ± s.e.m. of 6 mice. *p < 0.05, **p < 0.01, ***p < 0.001 versus vehicle-treated mice.

Given the important roles of FXR in NAFLD (Chao et al., 2016), we want to explore novel therapeutic effects of vidofludimus on NAFLD as an FXR ligand in addition to its anti-inflammatory function. The ob/ob mice produce a truncated inactive form of leptin and have been extensively studied as a model for NAFLD. Of note, no significant improvement of morbid obesity were observed by vidofludimus treatment in ob/ob mice (**Figure S6**). Excitingly, the vidofludimus treatment on ob/ob mice significantly reduced the levels of plasma triglycerides and cholesterol without affecting body weight (**Figures 6A–C**).

Serum cholesterol, including HDL and LDL/very LDL levels, was significantly decreased in ob/ob mice after treatment with vidofludimus. Histological examination and oil red O staining of liver sections obtained from vehicle-treated ob/ob mice showed the extensive existence of vesicular hepatocyte vacuolation, while vidofludimus treatment obviously reversed the liver from hepatic steatosis with the disappeared hepatic lipid accumulation and showed tight compact structure of the liver cells (**Figure 6D**), in consistent with the triglyceride levels in liver tissues (**Figure 6B**). The down-regulation of sterol regulatory element binding protein-1c (SREBP-1c) and carbohydrate responsive element binding protein (ChREBP), two major mediators of lipogenic gene expression (Dentin et al., 2005), in liver tissues of vidofludimus-treated mice (**Figure 6E**), further provided the molecular mechanisms of vidofludimus on hepatic steatosis amelioration in ob/ob mice. In addition to its potent anti-steatotic action, vidofludimus also suppressed the expression of inflammatory biomarkers in the liver (**Figure 6E**). Notably, the mRNA level of the FXR target genes FGF15, SHP, and iBABP in ileum, and organic solute transporter (OST) β in liver were all significantly upregulated in ob/ob mice administered with vidofludimus (**Figures 6E, F**), suggesting the effects of vidofludimus on NAFLD is through targeting FXR. Taken together, these results demonstrate that vidofludimus possesses remarkable beneficial effects in reducing hepatic steatosis and inflammation.

## Discussion

Vidofludimus is a novel oral immunomodulatory drug that has been carried out in Phase II trial to treat IBD and rheumatoid arthritis (RA) and in preclinical testing to treat lupus. Vidofludimus is a potent inhibitor of DHODH that is a key enzyme involved in the *de novo* pyrimidine biosynthesis (Fitzpatrick et al., 2010; Kulkarni et al., 2010), thus vidofludimus inhibits the proliferation of activated T and B cells *via* blocking DHODH (Leban et al., 2005). Vidofludimus also inhibits the expression of pro-inflammatory cytokines like IL-17 in activated lymphocytes, even in the presence of exogenous uridine, suggested a pharmacological eﬀect that was independent of inhibiting DHODH and T-cell proliferation (Fitzpatrick et al., 2012), however, of which the targeting mechanism remains unclear. In the present study, we found that vidofludimus inhibited the expression of IL-17 and other inflammatory cytokines including IL-6 and IL-1β, as well as COX-2 in the intestine from DSS-induced colitis mice is dependent on the existence of FXR. Crystallographic data authentically displayed that vidofludimus can bind in the ligand-binding pocket of FXR with a unique binding mode. Meanwhile, biochemical analysis proved that vidofludimus potently activated the FXR activity. In addition to cofactor recruitment assays, we performed two reporter systems (full-length system and pBind system) to detect the ligand-induced activity of nuclear receptors. The results showed that compared to the vehicle control, the activity of firefly luciferase to renilla luciferase ratio elevated about 3 times in the positive control in the FXR full-length system, and elevated about 30 times in the FXR pBind system, and both the firefly luciferase and the renilla luciferase reading were normal in these two groups in both COS-7 and 293T cells. While the firefly luciferase reading in cells treated with vidofludimus decreased sharply as the concentration increased from 1 to 5 µM, and the renilla luciferase reading were the same as positive and vehicle control. We hypothesize that vidofludimus may have some interference with Luciferase Assay Reagent II (LAR II) that quantifies the firefly luminescence. The phenomena in PPAR gamma reporter assay showed the same results, further supporting this hypothesis. In vivo mouse experiment further demonstrated that vidofludimus efficaciously improved DSS-induced colitis in WT mice but not FXR KO mice. These solid evidences indicate that in addition to DHODH, FXR is an important target for vidofludimus to exert its therapeutic effects on autoimmune disorders. Based on these results and the crucial roles of FXR in regulating NAFLD, repositioning of vidofludimus demonstrates that this potential IBD drug efficaciously ameliorated the NAFLD symptoms in ob/ob mice as expected, thus uncovering a novel disease indication and molecular basis for the drug development of vidofludimus. With the established safety and drug tolerability of vidofludimus in patients with IBD (Herrlinger et al., 2013), vidofludimus has potential to be a promising drug candidate for the treatment of NAFLD.

The efficacies of several agents in clinical trials, such as an FXR agonist (Ocaliva) (Neuschwander-Tetri et al., 2015; Younossi et al., 2019), CCR2 and CCR5 antagonists (Cenicriviroc) (Lalezari et al., 2011), and PPARα and PPARσ agonists (Elafibranor) (Ratziu et al., 2016), are encouraging based on liver biopsy or noninvasive markers, these clinical findings will need to translate into durable safety and efficacy. Pruritus and increased LDL cholesterol were the most commonly reported adverse events in obeticholic acid clinical studies (10 mg/25 mg oral dose of OCA once daily over a period of 18 months) (Younossi et al., 2019). According to recent paper from Yu. et al. (2019) bile acids (including CDCA) can work as natural ligands for human sensory neuron-expressed Mas-related G protein-coupled receptor X4 (MRGPRX4). Bile acids or other MRGPRX4 specific agonist induced itch in human subjects, providing a promising cause of OCA induced-pruritus (Meixiong et al., 2019a; Meixiong et al., 2019b; Yu et al., 2019). In addition, whether bile acid receptor TGR5 participates in cholestatic itch in human also remains unclear (Hodge et al., 2013; Cipriani et al., 2015). LDL-C is the low-density lipoprotein and the component of the cholesterol profile that causes the damage. Early increases in LDL cholesterol were observed in OCA treated patients who initiated with statins treatment. However, the initial LDL cholesterol increases reversed to below baseline levels as of month 6 and were sustained through month 18 (Younossi et al., 2019). In contrast, no drug-related serious adverse events were observed in vidofludimus clinical studies (35 mg oral dose of vidofludimus once daily over a period of 12 weeks), and Phase IIa ENTRANCE study met its primary endpoint in 88.5% of patients (complete and partial response). A total of 75 adverse events (AEs) were reported, of which 19 AEs were judged by investigators as “probably” drug-related. These included isolated cases of glucosuria, musculoskeletal pain, leucocyturia, nasopharyngitis, abdominal pain, fatigue, myalgia, microhematuria, tachycardia, insomnia, and dyspepsia. By comparing the clinical side effects of these two FXR ligands, vidofludimus appears to be a multi-target drug for IBD and NAFLD treatment, and the potential of DHODH inhibition in therapeutic effect and toxicity worth further exploration. The combination of two mechanisms of action provides an innovative therapeutic approach with broad clinical potential in various metabolic diseases, and the possibility of drug combinations as a future therapeutic option is increasingly likely because of concern that attacking a single target will not be sufficiently potent. In addition, the effects of vidofludimus on metabolism may be due to tissue-selective targeting of FXR in intestine in addition to liver, because vidofludimus showed similar or stronger regulation of FXR target genes in intestine (**Figure 6F**), compared to those in liver (**Figure 6E**). Although enteric FGF15 is shown to benefit hepatic steatosis (Fang et al., 2015), the role of drug-activated intestinal FXR in body physiology is poorly understood. Furthermore, many evidences have demonstrated that the transcriptional activation of nuclear receptors does not necessarily correlate with their biological effects (Gronemeyer et al., 2004; Picard et al., 2004; Higgins and Depaoli, 2010; Bokoch et al., 2010; Venkatanarayan et al., 2015). Administration of a full and potent FXR agonist, GW4064, resulted in weight gain and fat accumulation in mice (Watanabe et al., 2011), and even hepatobiliary injury to medaka eleutheroembryo (Howarth et al., 2010). In contrast, Our previously published data showed that doramectin, abamectin, and ivermectin as FXR partial agonists (Jin et al., 2013; Jin et al., 2015) have significant therapeutic effects on NAFLD in a relatively low dose. Accordingly, the FXR ligand vidofludimus in this study was characterized as partial agonist, different from classical full agonist.

Given the important roles of FXR in a variety of physiological and pathological processes, the ligands that regulate FXR activity are promising therapeutic agents for different diseases. Compared with many other nuclear receptors, the clinical applications of FXR ligands remain much less developed. Our findings reveal a promising template for the design of novel FXR ligands for the treatment of autoimmune disorders, such as IBD and RA. Moreover, the identification of FXR as a functional target for vidofludimus has not only provided novel mechanisms to optimize the compound for its immunomodulatory function but also uncovered its therapeutic effects on NAFLD based on the new established relationships among drugs, targets, and diseases.

## Data Availability Statement

The datasets generated for this study can be found in the PDB ID 5y1j.

## Ethics Statement

The animal study was reviewed and approved by the Institutional Animal Use and Care Committee of Xiamen University, Xiamen, Fujian 361102, China.

## Author Contributions

Participated in research design: LJ and YLi. Conducted experiments: YZ, YLu, SX, YJW, BY, FG, XZ, YMW, and YH. Performed data analysis: YZ, YLu, and LJ. Wrote or contributed to the writing of the manuscript: YZ, YLu, LJ, and YLi.

## Funding

This work was supported by grants from the National Natural Science Foundation of China (81773793, 31770814 and 81903691), the Fundamental Research Funds for the Central Universities (20720150052), the Programme of Introducing Talents of Discipline to Universities (B12001), the National Science Foundation of China for Fostering Talents in Basic Research (J1310027) and the Science and Technology Program of Xiamen (3502Z20194086).

## Conflict of Interest

The authors declare that the research was conducted in the absence of any commercial or financial relationships that could be construed as a potential conflict of interest.

## Abbreviations

NAFLD, non-alcoholic fatty liver disease; DHODH, dihydroorotate dehydrogenase; DSS, dextran sodium sulfate; FXR, farnesoid X receptor; NASH, non-alcoholic steatohepatitis; OCA, obeticholic acid; PBC, primary biliary cholangitis; PSC, primary sclerosing cholangitis; SRC, steroid receptor coactivator; NCoR, nuclear corepressor; CDCA, chenodeoxycholic acid; PPAR, peroxisome proliferator-activated receptor; RAR, retinoic acid receptor; RXR, retinoid X receptor; LXR, liver X receptor; AR, androgen receptor; ER, estrogen receptor; PXR, progesterone receptor; CAR, constitutive androstane receptor; LBD, ligand binding domain; IBD, inflammatory bowel disease; DAI, disease activity index; IL, interleukin; COX-2, prostaglandin-endoperoxide synthase 2; FGF15, fibroblast growth factor 15; SHP, small heterodimer partner; IBABP, ileal bile acid binding protein; AKR1B7, aldo-keto reductase 1b7; MPO, myeloperoxidase; TNBS, trinitrobenzene sulfonic acid; MEF, mouse embryonic fibroblast; MCP-1, monocyte chemotactic protein-1; CXCL2, C-X-C Motif Chemokine Ligand-2; NF-κB, nuclear factor κappa B; IκBα, NF-κB Inhibitor α; IKK α/β, Inhibitor Of NF-κB Kinase Subunit α/β; SREBP-1c, sterol regulatory element binding protein-1c; ChREBP, carbohydrate responsive element binding protein; OST, organic solute transporter; RA, rheumatoid arthritis.

## References

[B1] Akwabi-Ameyaw A. Bass J. Y. Caldwell R. D. Caravella J. A. Chen L. Creech K. L. (2009). FXR agonist activity of conformationally constrained analogs of GW 4064. Bioorg. Med. Chem. Lett. 19, 4733–4739. 10.1016/j.bmcl.2009.06.062 19586769

[B2] Bao J. Marathe B. Govorkova E. A. Zheng J. J. (2016). Drug Repurposing Identifies Inhibitors of Oseltamivir-Resistant Influenza Viruses. Angew. Chem. Int. Ed. Engl. 55, 3438–3441. 10.1002/anie.201511361 26833677 PMC4807618

[B3] Bokoch M. P. Zou Y. Rasmussen S. G. Liu C. W. Nygaard R. Rosenbaum D. M. (2010). Ligand-specific regulation of the extracellular surface of a G-protein-coupled receptor. Nature 463, 108–112. 10.1038/nature08650 20054398 PMC2805469

[B4] Carr R. M. Reid A. E. (2015). FXR agonists as therapeutic agents for non-alcoholic fatty liver disease. Curr. Atheroscler Rep. 17, 500. 10.1007/s11883-015-0500-2 25690590

[B5] Chao C. Y. Battat R. Al Khoury A. Restellini S. Sebastiani G. Bessissow T. (2016). Co-existence of non-alcoholic fatty liver disease and inflammatory bowel disease: A review article. World J. Gastroenterol. 22, 7727–7734. 10.3748/wjg.v22.i34.7727 27678354 PMC5016371

[B6] Cipriani S. Renga B. D’Amore C. Simonetti M. De Tursi A. A. Carino A. (2015). Impaired Itching Perception in Murine Models of Cholestasis Is Supported by Dysregulation of GPBAR1 Signaling. PloS One 10, e0129866. 10.1371/journal.pone.0129866 26177448 PMC4503431

[B7] Dentin R. Girard J. Postic C. (2005). Carbohydrate responsive element binding protein (ChREBP) and sterol regulatory element binding protein-1c (SREBP-1c): two key regulators of glucose metabolism and lipid synthesis in liver. Biochimie 87, 81–86. 10.1016/j.biochi.2004.11.008 15733741

[B8] Emsley P. Cowtan K. (2004). Coot: model-building tools for molecular graphics. Acta Crystallogr. D. Biol. Crystallogr. 60, 2126–2132. 10.1107/S0907444904019158 15572765

[B9] Fang S. Suh J. M. Reilly S. M. Yu E. Osborn O. Lackey D. (2015). Intestinal FXR agonism promotes adipose tissue browning and reduces obesity and insulin resistance. Nat. Med. 21, 159–165. 10.1038/nm.3760 25559344 PMC4320010

[B10] Fitzpatrick L. R. Deml L. Hofmann C. Small J. S. Groeppel M. Hamm S. (2010). 4SC-101, a novel immunosuppressive drug, inhibits IL-17 and attenuates colitis in two murine models of inflammatory bowel disease. Inflammation Bowel. Dis. 16, 1763–1777. 10.1002/ibd.21264 20310011

[B11] Fitzpatrick L. R. Small J. S. Doblhofer R. Ammendola A. (2012). Vidofludimus inhibits colonic interleukin-17 and improves hapten-induced colitis in rats by a unique dual mode of action. J. Pharmacol. Exp. Ther. 342, 850–860. 10.1124/jpet.112.192203 22691298

[B12] Flatt B. Martin R. Wang T. L. Mahaney P. Murphy B. Gu X. H. (2009). Discovery of XL335 (WAY-362450), a highly potent, selective, and orally active agonist of the farnesoid X receptor (FXR). J. Med. Chem. 52, 904–907. 10.1021/jm8014124 19159286

[B13] Gadaleta R. M. van Erpecum K. J. Oldenburg B. Willemsen E. C. Renooij W. Murzilli S. (2011a). Farnesoid X receptor activation inhibits inflammation and preserves the intestinal barrier in inflammatory bowel disease. Gut 60, 463–472. 10.1136/gut.2010.212159 21242261

[B14] Gadaleta R. M. Oldenburg B. Willemsen E. C. Spit M. Murzilli S. Salvatore L. (2011b). Activation of bile salt nuclear receptor FXR is repressed by pro-inflammatory cytokines activating NF-kappaB signaling in the intestine. Biochim. Biophys. Acta 1812, 851–858. 10.1016/j.bbadis.2011.04.005 21540105

[B15] Gronemeyer H. Gustafsson J. A. Laudet V. (2004). Principles for modulation of the nuclear receptor superfamily. Nat. Rev. Drug Discovery 3, 950–964. 10.1038/nrd1551 15520817

[B16] Haczeyni F. Poekes L. Wang H. Mridha A. R. Barn V. Geoffrey Haigh W. (2017). Obeticholic acid improves adipose morphometry and inflammation and reduces steatosis in dietary but not metabolic obesity in mice. Obesity 25, 155–165. 10.1002/oby.21701 27804232 PMC5849463

[B17] Herrlinger K. R. Diculescu M. Fellermann K. Hartmann H. Howaldt S. Nikolov R. (2013). Efficacy, safety and tolerability of vidofludimus in patients with inflammatory bowel disease: the ENTRANCE study. J. Crohns Colitis 7, 636–643. 10.1016/j.crohns.2012.09.016 23078909

[B18] Higgins L. S. Depaoli A. M. (2010). Selective peroxisome proliferator-activated receptor gamma (PPARgamma) modulation as a strategy for safer therapeutic PPARgamma activation. Am. J. Clin. Nutr. 91, 267S–272S. 10.3945/ajcn.2009.28449E 19906796

[B19] Hodge R. J. Lin J. Vasist Johnson L. S. Gould E. P. Bowers G. D. Nunez D. J. (2013). Safety, Pharmacokinetics, and Pharmacodynamic Effects of a Selective TGR5 Agonist, SB-756050, in Type 2 Diabetes. Clin. Pharmacol. Drug Dev. 2, 213–222. 10.1002/cpdd.34 27121782

[B20] Howarth D. L. Law S. H. Law J. M. Mondon J. A. Kullman S. W. Hinton D. E. (2010). Exposure to the synthetic FXR agonist GW4064 causes alterations in gene expression and sublethal hepatotoxicity in eleutheroembryo medaka (Oryzias latipes). Toxicol. Appl. Pharmacol. 243, 111–121. 10.1016/j.taap.2009.11.022 19963001 PMC2853180

[B21] Hu Y. Lounkine E. Bajorath J. (2014). Many approved drugs have bioactive analogs with different target annotations. AAPS J. 16, 847–859. 10.1208/s12248-014-9621-8 24871342 PMC4070249

[B22] Jin L. Li Y. (2010). Structural and functional insights into nuclear receptor signaling. Adv. Drug Delivery Rev. 62, 1218–1226. 10.1016/j.addr.2010.08.007 PMC2991384 20723571

[B23] Jin L. Feng X. Rong H. Pan Z. Inaba Y. Qiu L. (2013). The antiparasitic drug ivermectin is a novel FXR ligand that regulates metabolism. Nat. Commun. 4, 1937. 10.1038/ncomms2924 23728580

[B24] Jin L. Wang R. Zhu Y. Zheng W. Han Y. Guo F. (2015). Selective targeting of nuclear receptor FXR by avermectin analogues with therapeutic effects on nonalcoholic fatty liver disease. Sci. Rep. 5, 17288. 10.1038/srep17288 26620317 PMC4664883

[B25] Krishnan V. Ma Y. F. L. Chen C. Z. Thorne N. Bullock H. Tawa G. (2018). Repurposing a novel parathyroid hormone analogue to treat hypoparathyroidism. Br. J. Pharmacol. 175, 262–271. 10.1111/bph.14028 28898923 PMC5758387

[B26] Kulkarni O. P. Sayyed S. G. Kantner C. Ryu M. Schnurr M. Sardy M. (2010). 4SC-101, A Novel Small Molecule Dihydroorotate Dehydrogenase Inhibitor, Suppresses Systemic Lupus Erythematosus in MRL-(Fas)lpr Mice. Am. J. Pathol. 176, 2840–2847. 10.2353/ajpath.2010.091227 20413687 PMC2877845

[B27] Lalezari J. Gathe J. Brinson C. Thompson M. Cohen C. Dejesus E. (2011). Safety, efficacy, and pharmacokinetics of TBR-652, a CCR5/CCR2 antagonist, in HIV-1-infected, treatment-experienced, CCR5 antagonist-naive subjects. J. Acquir. Immune Defic. Syndr. 57, 118–125. 10.1097/QAI.0b013e318213c2c0 21317794

[B28] Leban J. Kralik M. Mies J. Gassen M. Tentschert K. Baumgartner R. (2005). SAR, species specificity, and cellular activity of cyclopentene dicarboxylic acid amides as DHODH inhibitors. Bioorg. Med. Chem. Lett. 15, 4854–4857. 10.1016/j.bmcl.2005.07.053 16143532

[B29] Lefebvre P. Staels B. (2014). Failing FXR expression in the liver links aging to hepatic steatosis. J. Hepatol. 60, 689–690. 10.1016/j.jhep.2014.01.001 24418014

[B30] Lefebvre P. Cariou B. Lien F. Kuipers F. Staels B. (2009). Role of Bile Acids and Bile Acid Receptors in Metabolic Regulation. Physiol. Rev. 89, 147–191. 10.1152/physrev.00010.2008 19126757

[B31] Lu Y. Ma Z. Zhang Z. Xiong X. Wang X. Zhang H. (2014). Yin Yang 1 promotes hepatic steatosis through repression of farnesoid X receptor in obese mice. Gut 63, 170–178. 10.1136/gutjnl-2012-303150 23348961

[B32] Meixiong J. Vasavda C. Green D. Zheng Q. Qi L. Kwatra S. G. (2019a). Identification of a bilirubin receptor that may mediate a component of cholestatic itch. Elife 8, e44116. 10.7554/eLife.44116 30657454 PMC6368403

[B33] Meixiong J. Vasavda C. Snyder S. H. Dong X. (2019b). MRGPRX4 is a G protein-coupled receptor activated by bile acids that may contribute to cholestatic pruritus. Proc. Natl. Acad. Sci. U. S. A 116, 10525–10530. 10.1073/pnas.1903316116 31068464 PMC6535009

[B34] Mencarelli A. Renga B. Migliorati M. Cipriani S. Distrutti E. Santucci L. (2009). The bile acid sensor farnesoid X receptor is a modulator of liver immunity in a rodent model of acute hepatitis. J. Immunol. 183, 6657–6666. 10.4049/jimmunol.0901347 19880446

[B35] Merk D. Sreeramulu S. Kudlinzki D. Saxena K. Linhard V. Gande S. L. (2019). Molecular tuning of farnesoid X receptor partial agonism. Nat. Commun. 10, 2915. 10.1038/s41467-019-10853-2 31266946 PMC6606567

[B36] Mi L. Z. Devarakonda S. Harp J. M. Han Q. Pellicciari R. Willson T. M. (2003). Structural basis for bile acid binding and activation of the nuclear receptor FXR. Mol. Cell 11, 1093–1100. 10.1016/S1097-2765(03)00112-6 12718893

[B37] Mudaliar S. Henry R. R. Sanyal A. J. Morrow L. Marschall H. U. Kipnes M. (2013). Efficacy and safety of the farnesoid X receptor agonist obeticholic acid in patients with type 2 diabetes and nonalcoholic fatty liver disease. Gastroenterology 145, 574–82 e1. 10.1053/j.gastro.2013.05.042 23727264

[B38] Neuschwander-Tetri B. A. Loomba R. Sanyal A. J. Lavine J. E. Van Natta M. L. Abdelmalek M. F. (2015). Farnesoid X nuclear receptor ligand obeticholic acid for non-cirrhotic, non-alcoholic steatohepatitis (FLINT): a multicentre, randomised, placebo-controlled trial. Lancet 385, 956–965. 10.1016/S0140-6736(14)61933-4 25468160 PMC4447192

[B39] Otwinowski Z. Minor W. (1997). Processing of X-ray diffraction data collected in oscillation mode. Methods Enzymol. 276, 307–326. 10.1016/S0076-6879(97)76066-X 27754618

[B40] Pappachan J. M. Babu S. Krishnan B. Ravindran N. C. (2017). Non-alcoholic Fatty Liver Disease: A Clinical Update. J. Clin. Transl. Hepatol. 5, 384–393. 10.14218/JCTH.2017.00013 29226105 PMC5719196

[B41] Pellicciari R. Fiorucci S. Camaioni E. Clerici C. Costantino G. Maloney P. R. (2002). 6alpha-ethyl-chenodeoxycholic acid (6-ECDCA), a potent and selective FXR agonist endowed with anticholestatic activity. J. Med. Chem. 45, 3569–3572. 10.1021/jm025529g 12166927

[B42] Pellicciari R. Passeri D. De Franco F. Mostarda S. Filipponi P. Colliva C. (2016). Discovery of 3alpha,7alpha,11beta-Trihydroxy-6alpha-ethyl-5beta-cholan-24-oic Acid (TC-100), a Novel Bile Acid as Potent and Highly Selective FXR Agonist for Enterohepatic Disorders. J. Med. Chem 59, 9201–9214. 10.1021/acs.jmedchem.6b01126 27652492

[B43] Picard F. Kurtev M. Chung N. Topark-Ngarm A. Senawong T. Machado De Oliveira R. (2004). Sirt1 promotes fat mobilization in white adipocytes by repressing PPAR-gamma. Nature 429, 771–776. 10.1038/nature02583 15175761 PMC2820247

[B44] Ratziu V. Harrison S. A. Francque S. Bedossa P. Lehert P. Serfaty L. (2016). Elafibranor, an Agonist of the Peroxisome Proliferator-Activated Receptor-alpha and -delta, Induces Resolution of Nonalcoholic Steatohepatitis Without Fibrosis Worsening. Gastroenterology 150, 1147–1159 e5. 10.1053/j.gastro.2016.01.038 26874076

[B45] Ratziu V. (2015). Starting the battle to control non-alcoholic steatohepatitis. Lancet 385, 922–924. 10.1016/S0140-6736(14)62010-9 25468161

[B46] Sepe V. Distrutti E. Fiorucci S. Zampella A. (2018). Farnesoid X receptor modulators 2014-present: a patent review. Expert Opin. Ther. Pat. 28, 351–364. 10.1080/13543776.2018.1459569 29649907

[B47] Sinal C. J. Tohkin M. Miyata M. Ward J. M. Lambert G. Gonzalez F. J. (2000). Targeted disruption of the nuclear receptor FXR/BAR impairs bile acid and lipid homeostasis. Cell 102, 731–744. 10.1016/S0092-8674(00)00062-3 11030617

[B48] Tully D. C. Rucker P. V. Chianelli D. Williams J. Vidal A. Alper P. B. (2017). Discovery of Tropifexor (LJN452), a Highly Potent Non-bile Acid FXR Agonist for the Treatment of Cholestatic Liver Diseases and Nonalcoholic Steatohepatitis (NASH). J. Med. Chem. 60, 9960–9973. 10.1021/acs.jmedchem.7b00907 29148806

[B49] Vavassori P. Mencarelli A. Renga B. Distrutti E. Fiorucci S. (2009). The bile acid receptor FXR is a modulator of intestinal innate immunity. J. Immunol. 183, 6251–6261. 10.4049/jimmunol.0803978 19864602

[B50] Venkatanarayan A. Raulji P. Norton W. Chakravarti D. Coarfa C. Su X. (2015). IAPP-driven metabolic reprogramming induces regression of p53-deficient tumours in vivo. Nature 517, 626–630. 10.1038/nature13910 25409149 PMC4312210

[B51] Wang Y. D. Chen W. D. Wang M. Yu D. Forman B. M. Huang W. (2008). Farnesoid X receptor antagonizes nuclear factor kappaB in hepatic inflammatory response. Hepatology 48, 1632–1643. 10.1002/hep.22519 18972444 PMC3056574

[B52] Wang M. H. Shim J. S. Li R. J. Dang Y. J. He Q. L. Das M. (2014). Identification of an old antibiotic clofoctol as a novel activator of unfolded protein response pathways and an inhibitor of prostate cancer. Br. J. Pharmacol. 171, 4478–4489. 10.1111/bph.12800 24903412 PMC4209153

[B53] Watanabe M. Horai Y. Houten S. M. Morimoto K. Sugizaki T. Arita E. (2011). Lowering bile acid pool size with a synthetic farnesoid X receptor (FXR) agonist induces obesity and diabetes through reduced energy expenditure. J. Biol. Chem. 286, 26913–26920. 10.1074/jbc.M111.248203 21632533 PMC3143650

[B54] Xiao H. Li P. Li X. He H. Wang J. Guo F. (2017). Synthesis and Biological Evaluation of a Series of Bile Acid Derivatives as FXR Agonists for Treatment of NASH. ACS Med. Chem. Lett. 8, 1246–1251. 10.1021/acsmedchemlett.7b00318 29259742 PMC5733277

[B55] Xiong X. Wang X. Lu Y. Wang E. Zhang Z. Yang J. (2014). Hepatic steatosis exacerbated by endoplasmic reticulum stress-mediated downregulation of FXR in aging mice. J. Hepatol. 60, 847–854. 10.1016/j.jhep.2013.12.003 24333182

[B56] Yamamoto Y. Gaynor R. B. (2004). IkappaB kinases: key regulators of the NF-kappaB pathway. Trends Biochem. Sci. 29, 72–79. 10.1016/j.tibs.2003.12.003 15102433

[B57] Yang Z. X. Shen W. Sun H. (2010). Effects of nuclear receptor FXR on the regulation of liver lipid metabolism in patients with non-alcoholic fatty liver disease. Hepatol. Int. 4, 741–748. 10.1007/s12072-010-9202-6 21286345 PMC2994619

[B58] Younossi Z. M. Ratziu V. Loomba R. Rinella M. Anstee Q. M. Goodman Z. (2019). Obeticholic acid for the treatment of non-alcoholic steatohepatitis: interim analysis from a multicentre, randomised, placebo-controlled phase 3 trial. Lancet 394, 2184–2196. 10.1016/S0140-6736(19)33041-7 31813633

[B59] Yu H. Zhao T. Liu S. Wu Q. Johnson O. Wu Z. (2019). MRGPRX4 is a bile acid receptor for human cholestatic itch. Elife 8, e48431. 10.7554/eLife.48431 31500698 PMC6773440

[B60] Zhang Y. Lee F. Y. Barrera G. Lee H. Vales C. Gonzalez F. J. (2006). Activation of the nuclear receptor FXR improves hyperglycemia and hyperlipidemia in diabetic mice. Proc. Natl. Acad. Sci. U. States America 103, 1006–1011. 10.1073/pnas.0506982103 PMC1347977 16410358

