# Supplementary material for: Repositioning an Immunomodulatory Drug Vidofludimus as a Farnesoid X Receptor Modulator With Therapeutic Effects on NAFLD
Source: Front Pharmacol. 2020 May 14;11:590. doi: 10.3389/fphar.2020.00590 (PMC7240069; doi:10.3389/fphar.2020.00590)
Supplement: Supplementary file 1 [file Presentation_1.pdf]

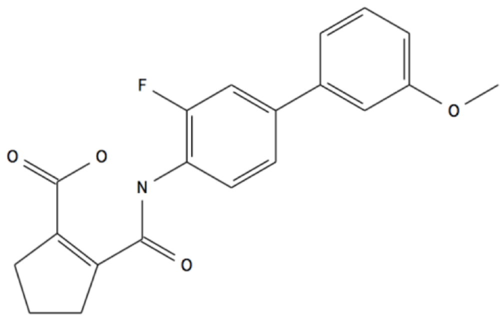

Vidofludimus

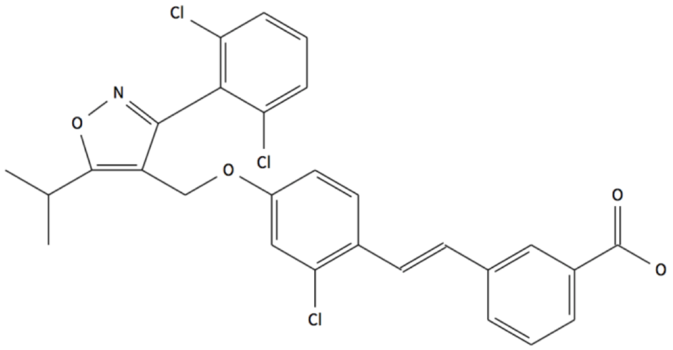

GW 4064

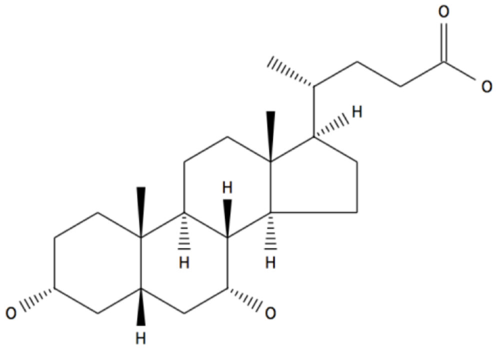

CDCA

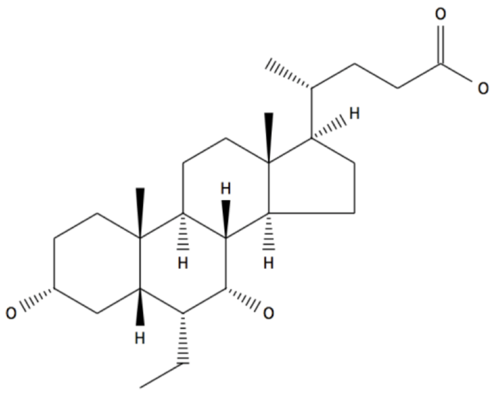

Obeticholic acid (OCA)

Figure S1. Chemical structures of vidofludimus and known FXR ligands. GW4064 is a synthetic high-affinity ligand for FXR, CDCA is a physiological low-affinity ligand, and OCA is a semisynthetic CDCA derivative with improved potency.

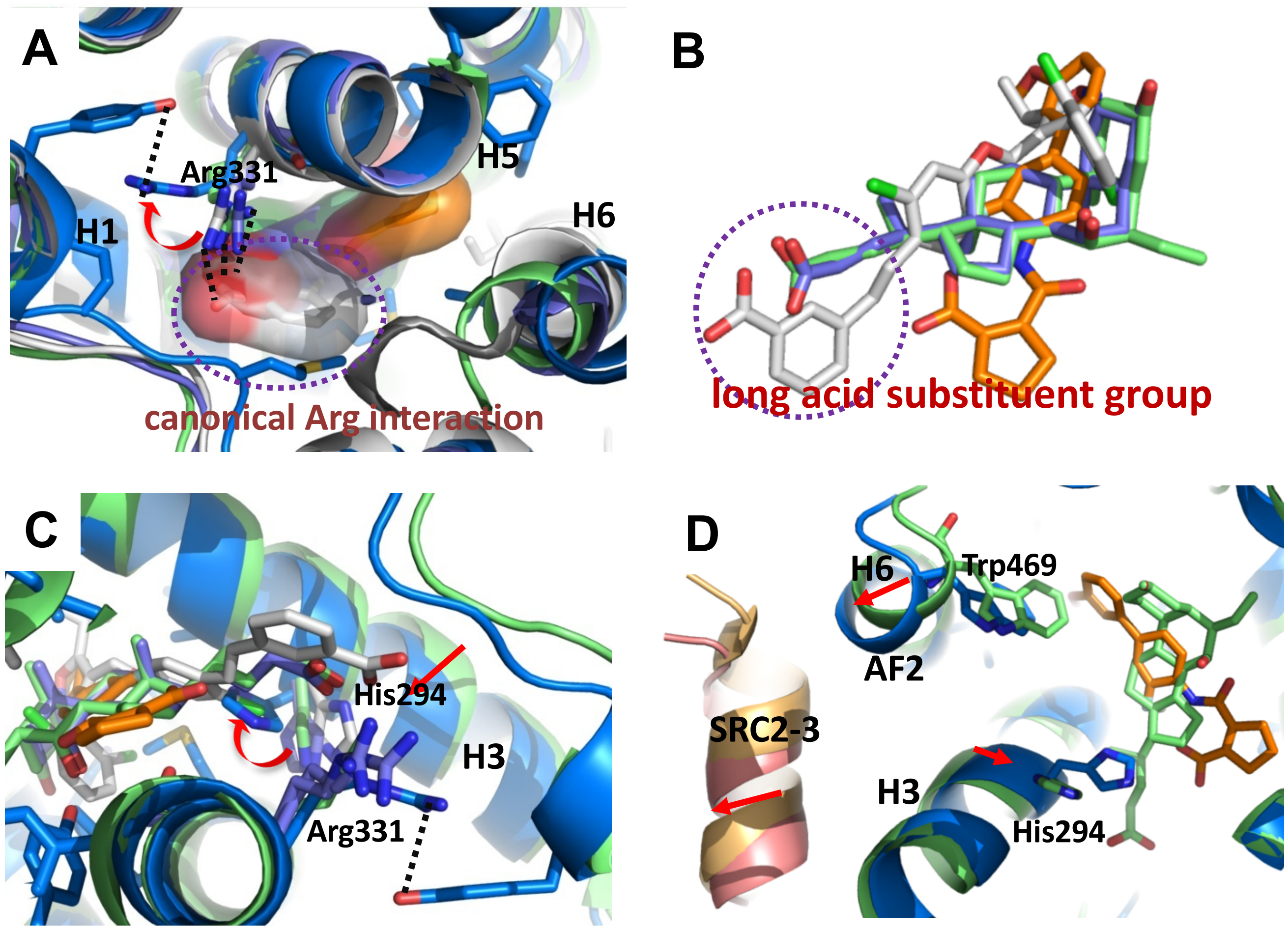

Figure S2. Structural comparison of the FXR-vidofludimus structure with the OCA-, GW4064-, and CDCA-FXR structures. (A, C) Alignment of the structure of vidofludimus-bound FXR LBD (PDB ID 5y1j, FXR is colored in dark blue) with OCA-bound FXR LBD (FXR is colored in light green), GW4064-bound FXR LBD (PDB ID 3dct, FXR is colored in white), and CDCA-bound FXR LBD (PDB ID 4qe6, FXR is colored in light purple), in cartoon representation. **(B)** Alignment of the four FXR ligands in their binding states. **(D)** Alignment of the structure of vidofludimus-bound FXR LBD (PDB ID 5y1j, FXR is colored in dark blue, and the SRC2-3 motif is in salmon) with OCA-bound FXR LBD (FXR is colered in light green and the SRC2-3 motif is in light orange).The conformation changes are indicated by red arrow, the hydrogen bond are indicated by dotted line. In (A-D), the bound ligands are shown in stick representation with carbon, nitrogen and oxygen atoms depicted in orange (vidofludimus), light green (OCA), white (GW4064) and light purple (CDCA).

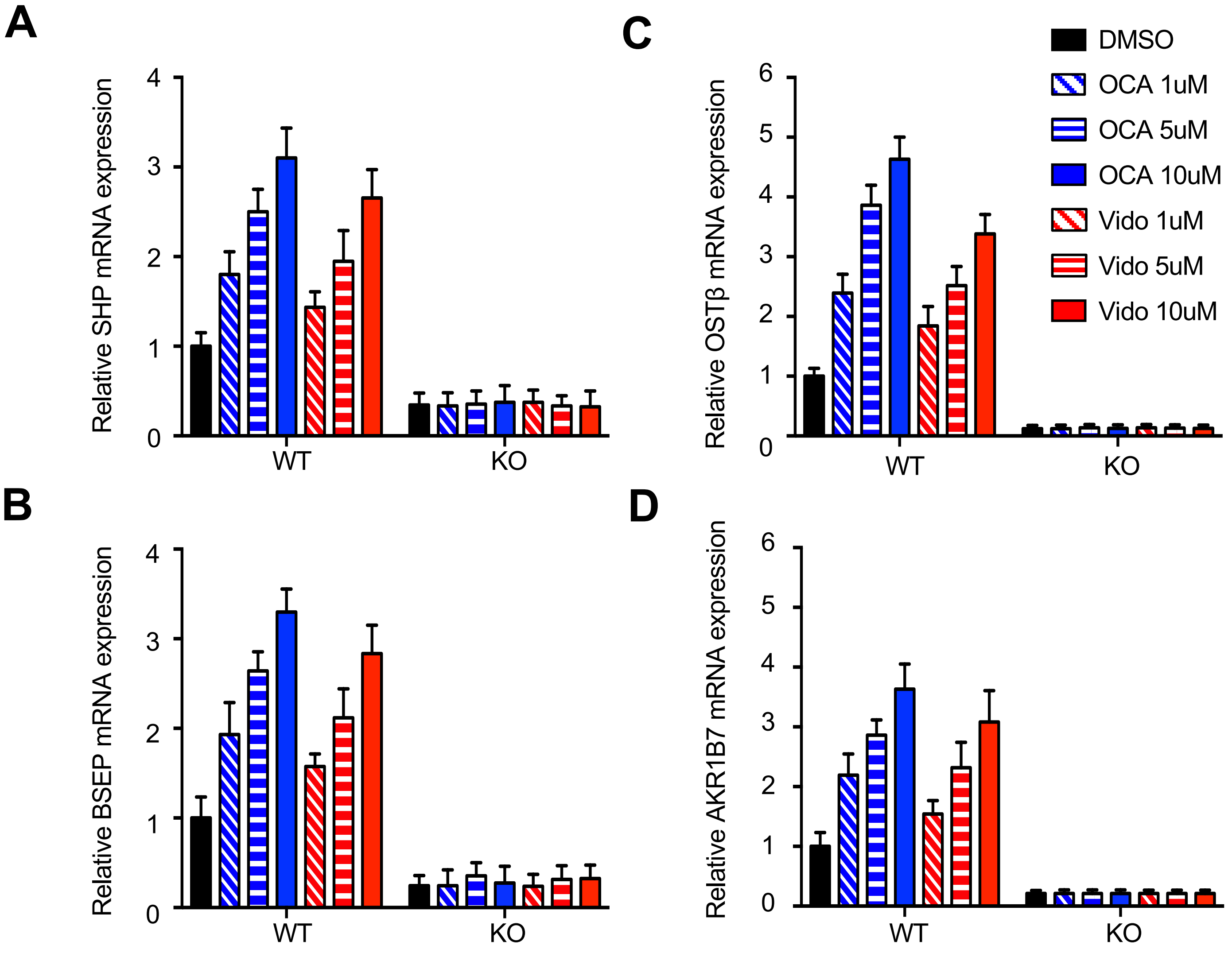

Figure S3. The relative mRNA levels of FXR target genes regulated by compounds in hepatocytes from mice. Primary hepatocytes were isolated from wild-type (WT) and FXR knock-out (KO) mice. After treated with three doses of OCA or vidofludimus for 24 hours, qPCR was performed to measure the mRNA levels of SHP, BSEP, OSTβ and AKR1B7. Relative mRNA levels were normalized to 36B4 levels. Values are the means ± s.e.m. of three independent experiments.

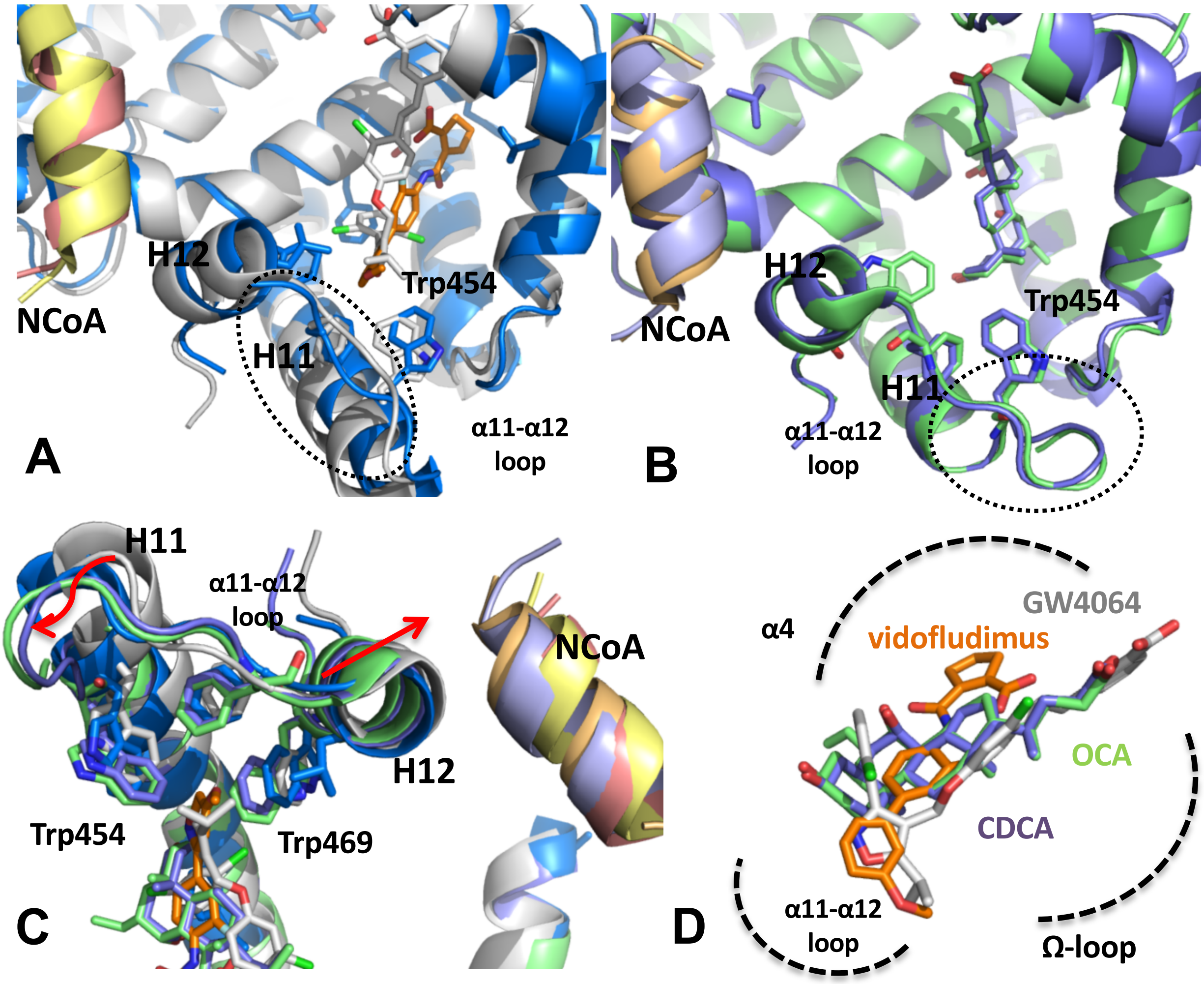

Figure S4. Structural comparison of the FXR-vidofludimus structure with the OCA-, GW4064-, and CDCA-FXR structures within the H11-H12 loop. (A) Alignment of the structures of vidofludimus-bound FXR LBD (PDB ID 5y1j, FXR is colored in dark blue) with GW4064-bound FXR LBD (PDB ID 3dct, FXR is colored in white). **(B)** Alignment of the structures OCA-bound FXR LBD (FXR is colored in light green) with CDCA-bound FXR LBD (PDB ID 4qe6, FXR is colored in light purple), in cartoon representation. **(C)** Alignment of the FXR-vidofludimus structure with the OCA-, GW4064-, and CDCA-FXR structures within the H11-H12 loop. The colors of various ligands bound FXR are the same as (A-B). The SRC2-3 motif for vidofludimus-FXR is colored in salmon; the NCoA motif for OCA-FXR is in light orange, for GW4064-FXR is in light yellow, and for CDCA-FXR is in light blue. The conformation changes are indicated by red arrows. **(D)** Alignment of the four FXR ligands in their binding states. In (A-D), the bound ligands are shown in stick representation with carbon, nitrogen and oxygen atoms depicted in orange (vidofludimus), light green (OCA), white (GW4064) and light purple (CDCA).

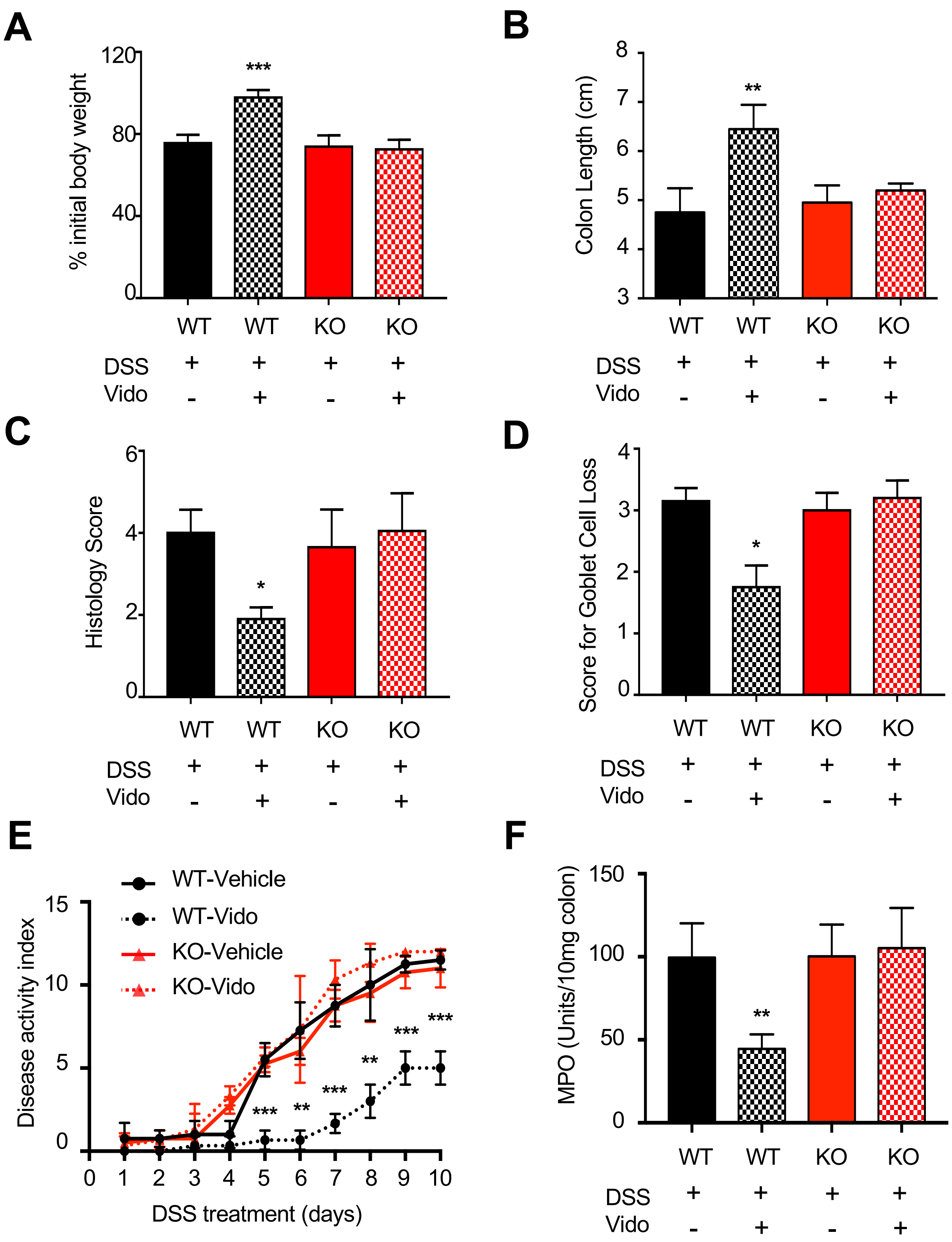

Figure S5. Vidofludimus ameliorates histological characteristics of DSS-induced colitis in an FXR dependent manner. (A) Percentage of body weight. (B) Colon length (cm) of wild-type (WT) and FXR-KO mice. (C) Histology score. (D) Score for goblet cell loss. (E) Disease activity index (DAI) score. (F) MPO (myeloperoxidase) activity. *$p<0.05$, **$p<0.01$, ***$p<0.001$ versus vehicle treated mice by one-way ANOVA with Dunn's post-test (n=6 per group).

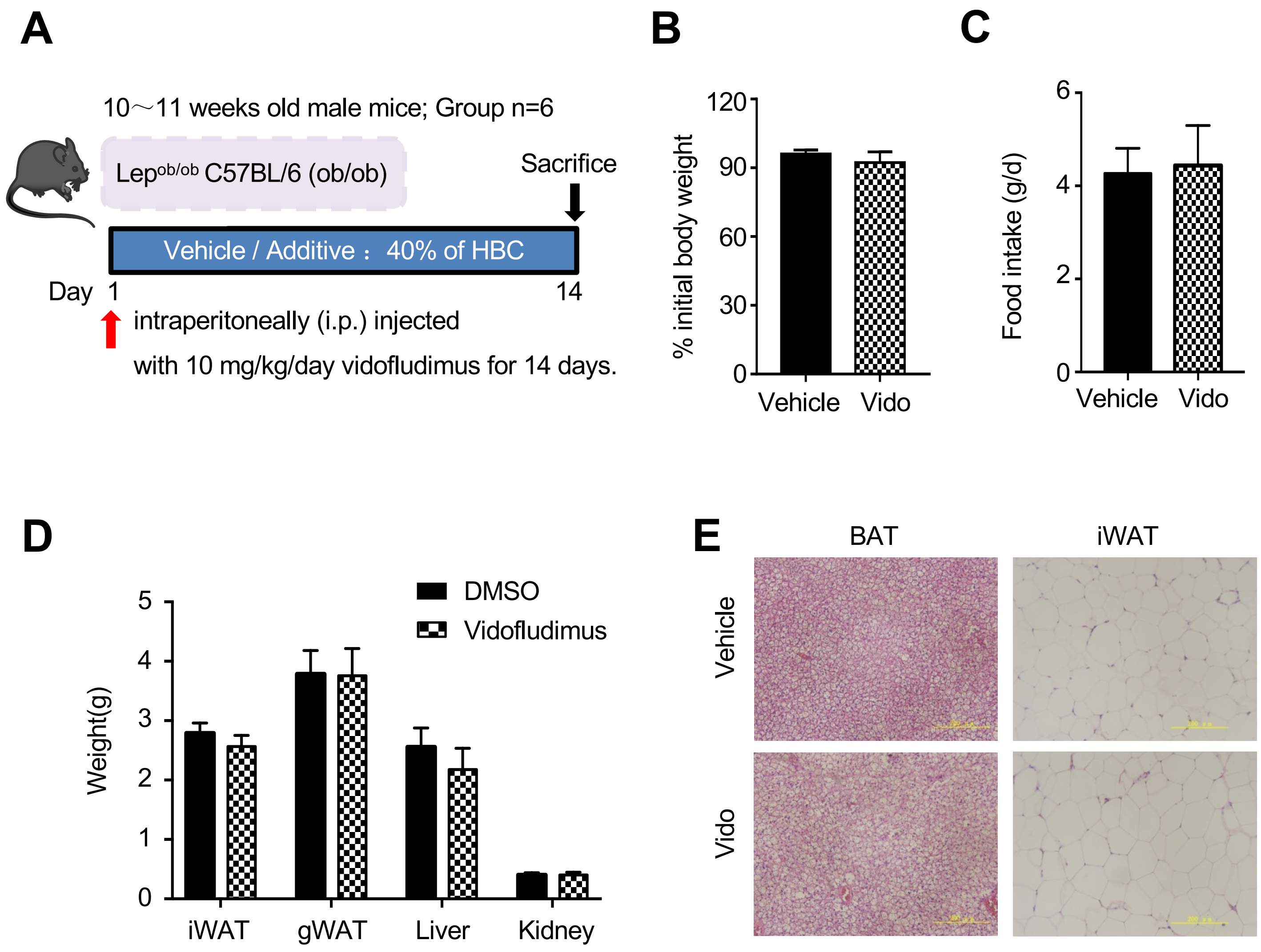

Figure S6. Vidofludimus treatment is ineffective for obesity by 14-day treatment in ob/ob mice. (A) Mice were treated with vehicle or vidofludimus for 14 days, n=6 per group. (B) Body weights. (C) Daily food intake. (D) Wet weight of inguinal WAT (iWAT), gonadal WAT (gWAT), liver and kidney. (E) Representative images for histological visualization of adipose sections stained with H&E. Scale bars, 200 um.

Table S1 X-ray crystallography: data collection and refinement statistics.

**Data collection**	**FXR/SRC2-3/Vidofludimus (PDB ID 5y1j)**		
Space group	C2221		
Cell dimensions			
a, b, c(Å)	34.67 162.05 115.62		
α,β,γ (°)	90.000 90.000 90.000		
Resolution (Å)	50-2.00 (2.03-2.00)		
R_{merge}	0.097 (0.413)		
No. of total/unique reflections	501390/22612		
I/sigmal	25.0(7.25)		
Completeness (%)	100.0(100.0)		
Redundancy	12.9(13.3)		
**Refinement:**	**Data Validation before publication**		
Resolution (Å)	47.06-2.00		
No. reflections	21448		
R_{work} / R_{free}	0.2089/0.2436		
No. atoms			
Protein	1853		
Ligand/ion	26		
Water	110		
B-factors			
Protein	25.559		
Ligand/ion	20.994		
Water	33.656		
R.m.s. deviations			
Bond lengths (Å)	0.023		
Bond angles (°)	2.021		
Ramachandran	Favoured	Allowed	Outliers
	99%	1%	0
Clash score	7		

Values in parenthesis are for highest-resolution shell.
R_{sym}=$\sum|I_i - \langle I \rangle|/\sum|I_i|$ where I_i is the intensity of the ith measurement, and $\langle I \rangle$ is the mean intensity for that reflection.
R_{work}=$\sum|F_{obs} - F_{calc}|/\sum|F_{obs}|$ where F_{obs} and F_{calc} are the observed and calculated structure factor amplitudes, respectively.
R_{free} is calculated using the same equation as that for R_{work} but 5% of reflections were chosen randomly and omitted from the refinement.

Table S2. Primers for qPCR.

Gene	Forward primer (5′-3′)	Reverse primer (5′-3′)
hB2M	GGCTATCCAGCGTACTCCAAA	CGGCAGGCATACTCATCTTTTT
hCXCL2	CCCATGGTTAAGAAAATCATCG	CTTCAGGAACAGCCACCAAT
hMCP-1	CAGCCAGATGCAATCAATGCC	TGGAATCCTGAACCCACTTCT
m36B4	CACTGGTCTAGGACCCGAGAAG	GGTGCCTCTGGAGATTTTCG
mIL-6	GAGGATTACCACTCCCAACAGACC	AAGTGCATCATCGTTGTTCATACA
mIL-1β	AGAGCATCCAGCTTCAAATC	GCTTCTCCACAGCCACAAT
mCOX2	TGAAAGCCCTCTACAGTGAC	GTGCTCCAAGCTCTACCAT
mIL17	CAGGACGCGCAAACATGA	GCAACAGCATCAGAGACACAGAT
mFGF15	GAGGACCAAAACGAACGAAATT	ACGTCCTTGATGGCAATCG
mSHP	GTCTTTCTGGAGCCTTGAGCTG	GTAGAGGCCATGAGGAGGATTC
mI-BABP	CAAGGCTACCGTGAAGATGGA	ACCTCCGAAGTCTGGTGATAGTTG
mOSTβ	GACAAGCATGTTCCTCCTGAGA	TGTCTTGTGGCTGCTTCTTTC
mCyp7a1	GCTTGTAGAGAGCCACACCAA	AGTGGTGGCAAAATTCCCA
mSREBP-1C	ATCGGCGCGGAAGCTGTCGG	GAAGTCACTGTCTTGGTTGTTG
mCHREBP	GCTCAACGCTGCCATCAAC	TGTCCCGCATCTGGTCAAA
mTNFa	GTAGCCCACGTCGTAGCAAAC	AGTTGGTTGTCTTTGAGATCCATG
mMCP-1	TCACCTGCTGCTACTCATTCACCA	TACAGCTTCTTTGGGACACCTGCT
mAKR1B7	CCACTGGCCACAGGGATT	TTTGCCTTTATTGTCTTTGGGTAA
